# Considerations for the Use of Machine Learning Extracted Real-World Data to Support Evidence Generation: A Research-Centric Evaluation Framework

**DOI:** 10.3390/cancers14133063

**Published:** 2022-06-22

**Authors:** Melissa Estevez, Corey M. Benedum, Chengsheng Jiang, Aaron B. Cohen, Sharang Phadke, Somnath Sarkar, Selen Bozkurt

**Affiliations:** 1 Flatiron Health, Inc., 233 Spring Street, New York, NY 10013, USA; mhedberg@flatiron.com (M.E.); corey.benedum@flatiron.com (C.M.B.); chengsheng.jiang@flatiron.com (C.J.); acohen@flatiron.com (A.B.C.); sharang.phadke@gmail.com (S.P.); ssarkar@flatiron.com (S.S.); 2 Department of Medicine, NYU Grossman School of Medicine, New York, NY 10016, USA

**Keywords:** artificial intelligence, deep learning, machine learning, oncology, personalized medicine

## Abstract

**Simple Summary:**

Many patient clinical characteristics, such as diagnosis dates, biomarker status, and therapies received, are only available as unstructured text in electronic health records. Obtaining this information for research purposes is a difficult and costly process, requiring trained clinical experts to manually review patient documents. Machine Learning techniques offer a promising solution for efficiently extracting clinically relevant information from unstructured text found in patient documents. However, the use of data produced with machine learning techniques for research purposes introduces unique challenges in assessing validity and generalizability to different cohorts of interest. To enable the effective and accurate use of such data for research purposes, we developed an evaluation framework to be utilized by model developers, data users, and other stakeholders. This framework can serve as a baseline to contextualize the quality, strengths, and limitations of using data produced with machine learning techniques for research purposes.

**Abstract:**

A vast amount of real-world data, such as pathology reports and clinical notes, are captured as unstructured text in electronic health records (EHRs). However, this information is both difficult and costly to extract through human abstraction, especially when scaling to large datasets is needed. Fortunately, Natural Language Processing (NLP) and Machine Learning (ML) techniques provide promising solutions for a variety of information extraction tasks such as identifying a group of patients who have a specific diagnosis, share common characteristics, or show progression of a disease. However, using these ML-extracted data for research still introduces unique challenges in assessing validity and generalizability to different cohorts of interest. In order to enable effective and accurate use of ML-extracted real-world data (RWD) to support research and real-world evidence generation, we propose a research-centric evaluation framework for model developers, ML-extracted data users and other RWD stakeholders. This framework covers the fundamentals of evaluating RWD produced using ML methods to maximize the use of EHR data for research purposes.

## 1. Introduction

Real-world data (RWD) leveraged from electronic health records (EHRs) can provide valuable insights into patients’ treatment pathways and facilitate health outcomes research. Specifically in oncology, where the treatment landscape is constantly evolving to include numerous targeted therapies for often rare populations of patients, RWD and real-world evidence (RWE) play a pivotal role in supplementing clinical trials [1,2,3] and the use of RWD to help clinicians, researchers, and other stakeholders in the healthcare ecosystem understand the ever-changing oncology treatment landscape is important. While some data elements are captured and stored in a structured format (e.g., lab results), the majority of data—including critical elements such as diagnosis dates, genomic test results, and adverse events—are captured and stored in unstructured (e.g., pathology reports, clinical narratives, etc.) formats [4]. Collecting these data from unstructured text has largely relied on manual chart review by an expert clinical abstractor, which can be challenging and time-intensive. Natural language processing (NLP) techniques address this challenge by providing automated solutions for processing free-text clinical notes to extract task-specific information, such as the metastasis status of a patient or adverse event to a treatment.

Today most of the NLP approaches use machine learning (ML) methods and have been applied across many clinical domains for a variety of information extraction tasks such as identifying a group of patients who have a specific diagnosis, share common characteristics, or show a progression of the disease including breast cancer, colorectal cancer, prostate cancer, and tuberculosis, etc. [3,5,6,7,8,9,10,11]. With the promises ML brings to RWD generation, in terms of scalability via reduced manual chart review burden [12,13,14], researchers and data vendors alike have already adopted ML to extract information documented in the EHR (ML-extracted RWD). Despite the increasing use of ML approaches for clinical information extraction, using these methods for research still introduces unique challenges, not only for specific tasks but also in terms of generalizability to different sub-cohorts.

Several interdisciplinary teams have proposed guidelines for developing and evaluating ML applications in healthcare [15,16,17,18,19,20]. These guidelines primarily focus on ML models that are intended to inform clinical decision-making at the point of care and often involve models that classify patients into different characteristics or predict future events (e.g., predicting a patient’s risk level or likelihood for a future event). As a result, these guidelines do not account for the unique considerations necessary for applying the use of ML-extracted data, collected from unstructured text in the EHRs, to health care outcomes research. Recent FDA draft guidance to assess RWD includes a section about using ML/AI for “unstructured data” in EHRs and encourages transparent reporting of the model and validation details [21]. However, the guidance does not include recommendations pertaining to the use of ML-extracted RWD from EHR for research purposes to generate RWE. The success of using ML-extracted RWD for research purposes (e.g., informing clinical development/trial design, regulatory decision making, etc.) broadly depends on its reliability, generalizability to cohorts of interest, and its fairness across demographic subgroups. Therefore, it is crucial to assess the performance of ML-extracted RWD for relevant clinical and demographic aspects to avoid harmful consequences which might systematically exclude some groups of patients [22]. Sendak et al. suggested including a “Warning” section for all clinical ML models developed for point of care [18] an approach that can be extended to ML-extracted RWD.

In order to enable successful, responsible, and effective use of ML-extracted RWD to support research for evidence generation, we created an evaluation framework for model developers, researchers using ML-extracted variables, and other stakeholders to address these unique considerations. This framework is intended only for RWD that is automatically extracted using ML methods from unstructured EHR documents [8,9,10] and examines model performance (e.g., sensitivity, PPV, and accuracy) as compared to chart abstraction (data abstracted by clinical experts) to the extent that human labeling is seen as a gold-standard. The goal of this framework is to cover the fundamentals of evaluating the performance, limitations, and fit-for-purpose use of ML-extracted RWD which can be used by different stakeholders for a variety of research purposes within the oncology landscape (e.g., breast and colorectal) as well as other clinical areas (e.g., tuberculosis, COVID-19) [3,5,6,7].

## 2. A Research-Centric Evaluation Framework for ML-Extracted RWD to Support Research and Evidence Generation

**Figure 1:**
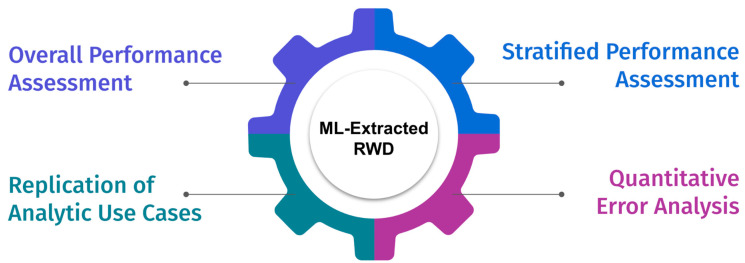
Evaluation Framework.

The research-centric evaluation framework consists of four modular components as shown in Figure 1. While each is an important component for assessing the appropriateness of the extracted RWD for analysis, the importance might vary based on the use case. This framework assumes that the model prototyping and validation steps are completed and documented using reporting best practices [19]. The intention of this evaluation framework is to be used as the first step before using ML-extracted RWD for research purposes.

### 2.1. Test Set

All components of the evaluation framework should be performed using a well-curated, representative dataset of an appropriate size to assess the generalizability of sub-cohorts derived from the population of interest [23,24,25,26,27,28]. This dataset, which is referred to as the “test set” here, should not be used during the model development (training and internal validation) process.

### 2.2. Overall Performance Assessment

Overall performance metrics (Table 1) aim to provide a high-level understanding of a given ML-extracted variable’s performance on the held-out test set described above. Performance metrics should help users make decisions based on the quality and the fit-for-use of an ML-extracted variable for common research purposes, such as cohort selection or retrospective analyses. It is also important to consider the clinical perspective while calculating and using these metrics. For example, while evaluating date variables, defining an error window in too narrow a way (example: 3 days) may not be clinically warranted for the use case and underestimate the performance of the date extraction model. Likewise using too large of an error window (example: 90 days) might be less clinically meaningful or trustworthy. This illustrates the importance of balancing performance metrics with the clinical utility to align on an error window that is appropriate for the specific use case.

### 2.3. Stratified Performance

Stratified performance analysis indicates whether model performance differs among certain subgroups (e.g., whether algorithmic bias exists) [29]. Stratified performance analysis can help indicate:

1. Whether model performance may be worse for a particular sub-cohort of interest given analyses often only cover specific sub-populations. For example, if recently diagnosed patients are of interest for an analysis, stratification by initial diagnosis year group can help detect whether there are any changes in documentation patterns (e.g., data shift) over time that is impacting model performance in the target population.
2. Whether model performance may be worse for a demographic group. High model performance does not preclude model errors from being concentrated in specific demographic groups due to bias introduced during model development or chance alone. Evaluating performance stratified by demographic subgroups, such as race and gender, may help to minimize unintentional discrimination caused by the model and ensure model fairness and generalizability. Readers should keep in mind that these assessments should also be performed during the validation process to ensure all actions needed to develop fair models were taken into account and at this stage, only the final findings are reported as confirmation or data use limitations.
3. Whether the performance is differential with respect to important covariates, such as treatment status, stage, biomarker status, and demographic characteristics, as many analytic use cases are focused on sub-cohorts that are receiving specific treatments. Differential error makes it harder to predict if and how model errors lead to bias in an analysis in which these variables are used as covariates.

Table 2 provides examples of stratifying variables that may be relevant for a cancer dataset and may not be applicable to other fields.

### 2.4. Quantitative Error Analysis

**Figure 2:**
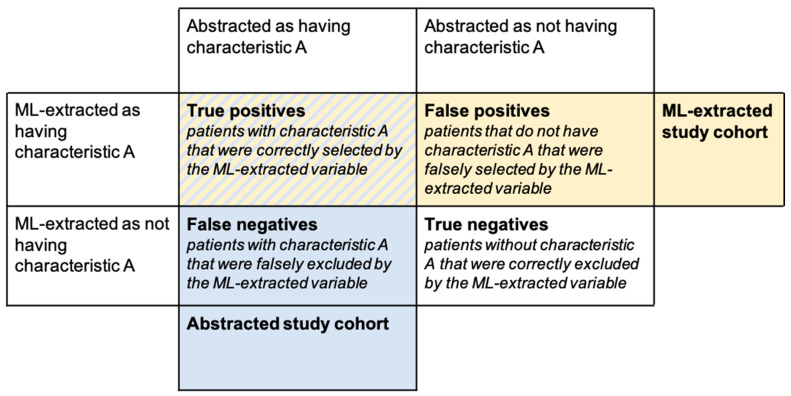
Confusion matrix for model errors.

Quantitative error analysis refers to the comparison of misclassified patients to correctly classified patients in terms of their demographic and clinical characteristics or outcomes. For example, let us assume there is a binary ML-extracted variable that is used to select a cohort of patients (e.g., we are interested in patients with characteristic A, where characteristic A is defined by an ML-extracted variable). Using the ML-extracted variable to define our study cohort, we select both true positives and false positives as the subjects for our analysis and incorrectly exclude false negatives (Figure 2) potentially biasing downstream analyses.

Through error analysis, we can learn how model errors impact what we observe in our selected study cohort and the potential biases introduced into downstream analyses by making the following comparisons in Table 3.

For each comparison mentioned above, we can look at a wide range of analyses. For example, we can compare demographic and clinical characteristics of patients correctly classified by the ML-extracted variable vs. misclassified. Similar to stratified performance metrics, this analysis can inform about a model’s fairness for intended use cases. We can also look directly at outcomes that are relevant for analyses of interest. In cancer research, common outcomes could include prevalence estimates, treatment patterns, and time-to-event analyses.

### 2.5. Replication of Analytic Use Cases

The goal of replication analyses is to provide insights into how the ML-extracted data perform on a breadth of use cases as compared to the corresponding abstracted variables. Replication analyses can range in complexity, from comparing cohorts (e.g., baseline characteristics and outcomes) selected using the ML-extracted variable vs. abstracted counterpart, to replicating analyses with multiple inclusion/exclusion criteria and complex statistical methodologies (e.g., comparative effectiveness studies or using ML-extracted data as a covariate or outcome variable). While it would not be practical to target an exact use case or all possible use cases, researchers should consider selecting a representative suite of analyses to enable stakeholders to evaluate whether a ML-extracted variable is fit for use for all anticipated use cases.

Model performance metrics such as sensitivity and PPV might not be informative enough to describe how analytical outcomes (such as real-world overall survival (rwOS) estimates or hazard ratios) may be impacted by model errors, as model errors may not be non-differential with respect to the outcome of interest and other relevant covariates. Moreover, conventional performance metrics are unable to describe how model errors interact with each other across multiple ML-extracted variables. Replication of analytic use cases can help contextualize the quality of model-extracted variables for end-use cases in several ways:

1. Replication of analytic use cases allows us to focus on specific sub-cohorts of interest.
2. The impact of model errors may differ based on how the variable is used. For example, a categorical variable can be used to select or stratify the study cohort, or it can be used as a variable in the analysis itself (e.g., as a covariate or in a propensity matching algorithm). A date variable can be used as the index date in a time-to-event analysis (e.g., rwOS from metastatic diagnosis date) or to select the study cohort (e.g., patients that started a particular therapy after metastatic diagnosis).
3. Model errors may be correlated rather than randomly distributed across the patient population. Replication analyses can shed light on the combined model performance which may be higher or lower for a selected cohort.
4. For event-level (rather than patient-level) variables, such as biomarker testing events, model performance metrics relevant to the actual use case can be difficult to define and interpret. For example, a biomarker model may over-predict the number of biomarker tests patients receive, resulting in lower test-level precision metrics. However if the majority of use cases are only interested in the biomarker test result closest to diagnosis, additional false-positive predictions at other temporal points would not introduce bias to the analysis results as long as the patient’s biomarker status at diagnosis is correctly predicted.

When evaluating ML-extracted variables through replication analysis, consideration should be given to the data available. If ML-extracted data are available without corresponding abstracted data for a cohort of interest, the analysis may be limited to replicating results published in the literature. While this comparison can indicate external validity, it will not be able to yield any insight as to how model errors may influence the reproduced result. For example, the published and reproduced result may differ despite high model performance simply because of differences in the study population. Conversely, if abstracted and ML-extracted data are available for the cohort of interest, researchers can design in silico experiments to understand how model errors may influence the parameter of interest. To evaluate the potential impact of model errors, sensitivity analyses, such as quantitative bias analysis, can be conducted by researchers to better understand whether model misclassification is systematically introducing bias [30].

## 3. Illustrative Use Case

A model that extracts metastatic diagnosis from the EHR will be used as an illustrative use case to demonstrate how the framework can be applied to a specific ML-extracted variable (Table 4). The ultimate purpose of the use case is to use the ML-extracted metastasis variable to select a study cohort for a health outcomes research question.

## 4. Discussion

While ML has tremendous promise for unlocking the power of RWD it is important to note these models are not always perfect and should be evaluated and monitored for any potential bias or findings to prevent negative consequences on the integrity of the data. For example, systematic misclassification errors for metastatic patients might cause the model to select a skewed cohort of patients and cause unrepresentative results. Therefore, it is critical to use a rigorous evaluation framework that assesses clinical ML applications early and at various stages of their development. However, currently available evaluation and reporting frameworks fall short in assessing ML-extracted RWD, despite growing and potential use cases of these data for research or regulatory decision-making [15,16,17,18,19]. Our goal was to put forth a framework for minimum evaluation standards necessary to understand the quality of ML-extracted RWD for target populations and to expose hidden biases of an information extraction tool for researchers. We believe that before using any ML-generated RWD for research or decision-making purposes, researchers must first check the strengths and limitations of these data sets through a standardized evaluation framework [33].

In addition to our proposed evaluation framework, we provided an example use case for a hypothetical metastatic variable. This use case allowed us to demonstrate the strengths and limitations of this variable which users can take into account while using the ML-extracted variable in their research studies. First, the metastatic variable had over 90% sensitivity and specificity. Second, while sensitivity was 5% higher for “Black or African American” race group vs. “White”, the PPV for the “metastatic” class was 5% lower, which can lead to a ML-selected metastatic cohort having a higher rate of Black or African American patients with false-positive metastatic classification relative to White patients. Third, the errors did not cause a difference in the example outcome analysis (rwOS). Fourth, the model had sufficient performance to demonstrate similar results with abstracted data when this variable was used to select a study-specific cohort from the target population.

Information extraction using ML enriches the potential utilization of EHR-derived unstructured text for quality improvement, clinical and translational research, and/or regulatory decision-making. Unsurprisingly the importance of automated information extraction applications has been growing throughout the RWD life cycle. While there are many suggestions and guidelines by scientists and regulators to assess the quality and fit-for-purpose of RWD [21,34,35], there is still a gap in evaluating ML-extracted RWD in terms of accuracy, bias, fairness, and error distributions. Moreover, while the use of ML and NLP can improve the power and coverage of RWD, they do not replace the need for a well-thought-out research question and longitudinal data sets.

For most analyses using RWD, multiple ML-extracted variables will be used in cohort selection or be statistically summarized. This “layering” of variables may result in end-to-end variable performance differing from the variable level performance assessment in the test set. For example, if a user is selecting a cohort of patients with ALK+ disease treated with Drug A, patients who are incorrectly classified as receiving Drug A (i.e., false positive) but correctly classified as not having ALK+ disease (i.e., true negative) will still be correctly excluded from this cohort. Thus, when we consider the precision of Drug A within this cohort, it would be higher than the precision measured within the performance assessment framework. While it is not possible to assess all use cases, replicating a set of common use cases including several ML-extracted variables might provide a better fit-for-purpose assessment of the data set created.

The evaluation framework we propose for ML-extracted variables is not without limitations. First, it does not include any thresholds of what level of performance is “good enough”. Hernandez-Boussard et al. (2019) [36] proposed a threshold for regulatory grade extracted data as recall >85% and precision >90%, however, this suggestion does not rely on an empirical study, and the authors aimed to propose a starting benchmark to initiate discussion. Since the minimum acceptable performance threshold may be use-case dependent and there is no commonly accepted definition of “good enough,” we believe the “replication of use case” component of our framework would provide the insights necessary for determining such thresholds for different research scenarios. Our framework can also be combined with other frameworks to assess Regulatory-Grade Data Quality [37] for regulatory use cases.

Second, the stratified performance assessment component of the framework suggests the evaluation of several variables of interest which might become challenging when deciding what action to take once all comparisons are completed. We encourage the users of the framework to select the subset of variables based on their use case and set acceptable error rates in advance of the evaluation process. While choosing the set of variables for this step, it would be beneficial to keep in mind the target cohort, clinical question, and seven sources of harm in ML [38].

Third, our framework does not include continuous model monitoring with data-in-the-wild (i.e., data that are currently unseen but could be encountered in the EHR in the future). For example, a new site could be added to the network of oncology clinics that was not there when the model was trained; thus, continuous monitoring of such models is an important piece to consider for ML-extracted data in recurring datasets. Fourth, our framework does not include investigations regarding the explainability of the models used [5], however, this framework can be extended to account for any advancement in this active field of research.

Finally, we developed and applied this evaluation framework to RWD harnessed for oncology use cases. However, we feel the components of this framework are important to understand when using models to extract variables, irrespective of the disease of interest, and should not be limited or restricted to oncology.

## 5. Conclusions

Innovations in machine learning offer new opportunities and solutions to accelerate health research, including the generation of real-world data at a scale previously unachievable through conventional manual chart reviews. This may provide immense value as demand increases for research into niche subpopulations that require larger RWD cohort sizes. While holding tremendous promise, machine learning solutions introduce unique challenges, such as access to high-quality data for model development, generalizability to cohorts of interest, and lack of model interpretability. To enable the widespread adoption of such methods, machine learning practitioners must describe the methods used to evaluate the model and the implication of model errors in downstream analyses. To this end, we proposed an evaluation framework for research-centric assessment of ML-extracted RWD before the data are used for research purposes. Our evaluation framework provides a structured approach to documenting the strengths, limitations, and applications of ML-extracted RWD. By going beyond standard machine learning metrics with the inclusion of stratified performance analysis, quantitative error analysis, and replication analysis, this framework enables a deeper understanding of potential model biases and how the ML-extracted data may perform in research use cases as compared to the corresponding abstracted variables. Despite its limitations, we feel that our proposed framework can be used as a baseline and with further testing, can be applied to additional use cases and disease types. Ultimately, our framework can be used to define a minimum quality threshold when using ML-extracted RWD for different use cases. As ML-extraction methods continue to advance, this framework can also be extended or modified to accommodate new variable types or uses of ML-extracted variables. We believe the adoption of such frameworks will improve transparency with respect to the quality of ML-extracted variables and can promote the responsible and effective use of ML-extracted RWD.

## Figures and Tables

**Table 1:** Example performance metrics.

Variable Type	Example ML-Extracted Variable	Example Performance Metric
Categorical	Diagnosis (yes/no)	Sensitivity Positive predictive value (PPV or precision) Specificity Negative predictive value (NPV) Accuracy Predicted prevalence vs. abstracted prevalence Calibration plots (if applicable)
Date	Diagnosis date	Sensitivity with a ± n-day window PPV with a ± n-day window ^1^ Distribution of date errors
Continuous	Lab value	Sensitivity, PPV, and accuracy for classifying the result as within vs. outside the normal range Sensitivity, PPV, and accuracy for classifying the result within ±X of the true value Mean absolute error (MAE)

^1^: The proportion of patients’ human-abstracted as having the diagnosis that is also correctly identified as having the diagnosis by the model and where the ML-extracted diagnosis date is within ±n days of the abstracted diagnosis date or both abstracted and ML-extracted dates are unknown.

**Table 2:** Stratified analysis steps and example variables.

Goal	Example Strata Variables
(I.) Understand performance in sub-cohorts of interest	Year of diagnosis (e.g., before vs. after year x) Treatment status (treated vs. not treated) Biomarker status (positive vs. negative)
(II.) Fairness	Race and ethnicity group Gender Age group Insurance status
(III.) Risk for statistical bias in analysis	Treatment setting (Academic vs. Community) Cancer stage at diagnosis Age at diagnosis Cancer histology Smoking status Treatment status (treated vs. not treated) Biomarker status (positive vs. negative)

**Table 3:** Examples for comparison of errors and their interpretation.

Comparison	Usefulness/Interpretation
True positives vs. False negatives	This comparison informs whether patients incorrectly excluded from the study cohort differ from those correctly included with respect to patient characteristics or outcomes. If true positive and false negative patients appear similar: Model misclassification may be random and excluded patients will most likely have a minimal impact on analysis results. If true positive and false negative patients appear different: Model misclassification may be systematic and excluded patients may impact analysis results.
True positives vs. False positives	This comparison informs whether patients incorrectly included in the study cohort differ from those correctly included with respect to patient characteristics or outcomes. If true positive and false positive patients appear similar: Model misclassification may be random and incorrectly included patients may have a minimal impact on analysis results. If true positive and false positive patients appear different: Model misclassification may be systematic and incorrectly including patients may impact analysis results.

**Table 4:** Evaluation framework template for the illustrative example.

**Variable:** Metastatic Diagnosis (yes/no)		
**Model Description**^1^ Inputs to the model include unstructured documents from the EHR (e.g., visit notes, pathology/radiology reports). The output of the model is a binary prediction (yes/no) for whether the patient has a metastatic diagnosis at any time in the record.		
**Target Dataset/Population** The model is used in a dataset that contains patients with non-small cell lung cancer (NSCLC).		
**Common Analytic Use Case** Selecting a cohort of patients who have (or do not have) metastatic disease Using metastatic status as a covariate or stratifying variable in an analysis		
**ML-Extracted Variable Evaluation**		
**Components**	**Description**	**Hypothetical Results and Findings**
Test Set	The size of the test set is selected to achieve a target margin of error for the primary evaluation metric (e.g, sensitivity or PPV) within the minority class (metastatic disease). To measure model performance, a random sample of patients is taken from a NSCLC cohort and withheld from model development.	Patients selected from the target population which is not included in model development
Overall Performance	As the primary use of this variable is to select a cohort of metastatic patients, sensitivity, PPV, specificity, and NPV are measured. To evaluate how well this variable selects a metastatic cohort, emphasis is placed on sensitivity and PPV to understand the proportion of patients missed and the proportion of patients incorrectly included in the final cohort.	Sensitivity ^2^ = 0.94 PPV ^3^ = 0.91 Specificity ^4^ = 0.90 NPV ^5^ = 0.90
Stratified Performance	Sensitivity and PPV for both Metastatic and Non-metastatic classes are calculated across strata of variables of interest. Stratifying variables are selected with the following goals in mind: Performance in sub-cohorts of interest (e.g., year of diagnosis) Fairness (e.g., race and ethnicity) Risk for statistical bias in analysis (e.g., cancer stage at diagnosis)	Example finding for race and ethnicity: Sensitivity for the “metastatic” class is 5% better for “Black or African American” race group vs. “White”. PPV for the “metastatic” class is 5% lower for “Black or African American” race group vs. “White”
Quantitative Error Analysis	To understand the impact of model errors on the selected study cohort, baseline characteristics and rwOS are evaluated for the following groups True positives vs. false negatives True positives vs. false positives Typically, patients with non-metastatic disease have longer survival times than patients with metastatic disease. If model misclassification is random, the inclusion of false positives in the study cohort will result in longer observed survival times. However, if model misclassification is systematic and false positives have survival similar to patients with metastatic disease, then the distribution of survival times may remain relatively unchanged.	Example findings from rwOS analysis *: rwOS ** for False Positives (21 months) was similar to True Positives (17 months). Example findings from baseline characteristic analysis: Compared to true negatives, false positives are less likely to have a history of smoking (86% vs. 91%).
Replication of Use Cases	Evaluate rwOS from metastatic diagnosis date for patients selected as metastatic by the ML-extracted variable vs. abstracted counterpart (outcomes in the general population)	rwOS for ML extracted cohort: 9.8 months (95% CI 8.92–10.75) rwOS for abstracted cohort: 9.8 months (95% CI 8.92–10.69)

^1^: Model is constructed using snippets of text around key terms related to “metastasis,” and processed by a long short-term memory (LSTM) network to produce a compact vector representation of each sentence. These representations were then processed by additional network layers to produce a final metastatic status prediction [31]. ^2^: Sensitivity refers to the proportion of patients abstracted as having a value of a variable (e.g., metastasis = true) that are also ML-extracted as having the same value. ^3^: PPV refers to the proportion of patients ML-extracted as having a value of a variable (e.g., metastasis = true) that is also abstracted as having the same value. ^4^: Specificity refers to the proportion of patients abstracted as not having a value of a variable (e.g., metastasis = false) that are also ML-extracted as not having the same value. ^5^: NPV refers to the proportion of patients ML-extracted as not having a value of a variable (e.g., metastasis = false) that are also abstracted as not having the same value. *: rwOS analysis was performed using Kaplan–Meier method [32]. **: The index date selected for rwOS calculation can be changed based on the study goals. However, the index date that is selected should be available for all patients, regardless of the concordance of their abstracted and predicted value. In this illustrative example, we provided the rwOS strictly as an example and do not specify the index date as index date selection will be case-dependent.

## Data Availability

The simulated data used in this study have been originated by Flatiron Health, Inc. These simulated de-identified data may be made available upon request and are subject to a license agreement with Flatiron Health; interested researchers should contact <DataAccess@flatiron.com> to determine licensing terms.
